# Linguistic and non-linguistic cues in motion event endpoint description: The selection between English *to* and *towards*

**DOI:** 10.3758/s13421-022-01371-6

**Published:** 2022-11-16

**Authors:** Yiyun Liao, Katinka Dijkstra, Rolf A. Zwaan

**Affiliations:** Department of Psychology, Education and Child Studies, Erasmus University Rotterdam, P.O. Box 1738, 3000 DR Rotterdam, the Netherlands

**Keywords:** The actor’s goal, The interlocutor’s social status, Grammatical aspect, Directional prepositions, Motion event endpoint description

## Abstract

The current study aims to explore the factors that could affect people’s description of a motion event endpoint. The study conducted by Liao, Dijkstra, and Zwaan (2021, *Language and Cognition*, *13*[2], 161–190) found that two non-linguistic factors (i.e., the actor’s goal and the interlocutor’s social status) affect people’s choice between two Dutch directional prepositions (i.e., *naar* and *richting*) during event description tasks. The current study aims to extend these findings by examining the choice between a similar pair of directional prepositions in English (i.e., *to* and *towards*). Moreover, we aim to study whether grammatical aspect (i.e., the English simple present and the English progressive aspect) affects the sensitivity to the two non-linguistic factors and consequently also affects how people describe a motion event endpoint. In Experiment 1, we used the English simple present for all sentence stimuli (e.g., *he walks (?) the trash bin*). We found a significant effect of Interlocutor (the interlocutor’s social status) on preposition choice, but no significant effect of Intention (the actor’s goal). In Experiment 2, we replaced the English simple present with the English progressive aspect (e.g., *he is walking (?) the trash bin*). We found significant main effects of both Interlocutor and Intention on preposition choice. These findings extend those reported in Liao et al. (2021) *Language, Cognition and Neuroscience*, 35(4), 498–520 in that protagonist intention and interlocutor status were found to indeed affect motion event endpoint description. The current findings furthermore show that grammatical aspect affects people’s sensitivity to these factors, thus also affecting how a motion event endpoint is described.

## Introduction

To describe an event in words, we first need to process all the relevant information about the event and then decide what message we want to convey to achieve a certain communicative purpose. These processes of message planning before the formation of utterances are called the conceptualization phase (Levelt, 1989, 1999). Moreover, to convey these messages through language, we also need to choose what word and grammar within the language of utterance are the most suitable according to the current situation. Hence, event description is a combination of event conceptualization and linguistic constraints (grammar and word choice).

In many motion event description and conceptualization studies, converging evidence shows that the endpoint of a motion event is more salient than the source of a motion event (e.g., Lakusta & Carey, 2015; Lakusta & Landau, 2005, 2012; Papafragou, 2010; Regier & Zheng, 2007). Specifically, endpoints are mentioned more often (e.g., *the bird flew* ***to a tree***) than sources (e.g., *the bird flew* ***from a signpost***) in motion event descriptions (e.g., Papafragou, 2010). Endpoints are also remembered better than sources after the description task (e.g., Lakusta & Landau, 2012). Many studies have discussed this endpoint-bias phenomenon and the reasons why it occurs (e.g., Johanson et al., 2019; Papafragou, 2010). Hence, we do not elaborate on this matter any further. In the current study, we are particularly interested in the factors that might affect how people describe a motion event endpoint, given its more salient status compared with other motion event components.

The first factor that we find relevant and important to the identification of a motion event endpoint during motion event description is the intention of the moving agent. Anticipating upcoming information and predicting the near future is a fundamental part of our daily life. Given that human actions are often goal directed, knowing the goal of the actor can greatly help the observer to understand an ongoing activity by predicting its possible endpoint (Zacks, 2004). For instance, if you see that your sister is drawing and you know that your sister wants to draw a house, you will then not expect her to stop after she just finished drawing a roof. A predicted endpoint of her drawing activity will be a complete picture of a house. Studies have shown that the ability to infer an event endpoint from the actor’s goal has already been found in infants (Baldwin et al., 2001).

In motion event studies, researchers have also discovered a strong connection between the animacy/intentionality of the agent and the memory of the event endpoint during event cognition (Lakusta & Carey, 2015; Lakusta & Landau, 2012). However, most of these studies focus on comparing animate agents with inanimate agents. Not much attention has been paid to comparing animate agents, such as the extent to which the presence/absence of a clear goal of an animate agent affects the construal of a motion event endpoint. Therefore, in the current study, we would like to investigate this aspect. We expect to find a strong connection between the actor’s goal and the identification of a motion event endpoint during event description.

Another factor that we consider important during the process of event description is the formality of the speech context. As proposed by Heylighen and Dewaele (2002), there are four parameters that determine the degree of formality in speech context—namely, the speech topic, the setting, the speech modality (written vs. spoken), and the interlocutor. In the current study, we are especially interested in examining the effect of the social status of the interlocutor (i.e., the social distance between the speaker and the interlocutor) on motion event endpoint description. As social animals, we care about the social status of our audience/our interlocutor. Many studies have provided evidence that speakers accommodate their speech in correspondence to the knowledge of their interlocutor to achieve successful communication (accommodation theory; Giles & Powesland, 1975; Giles & Smith, 1979). However, in most of the motion event description studies, the effect of the interlocutor is often overlooked and is certainly understudied.

Event description rarely happens in isolation. An audience or an interlocutor is usually involved. Regarding event endpoint construal, it is probable that an event endpoint will be defined differently depending on the social distance between the speaker and the interlocutor. Just take a simple daily event as an example: cleaning dishes. Depending on who is listening: their mom or an exacting manager of a three-star Michelin restaurant, speakers might even define the endpoint of cleaning dishes differently. In the latter case, the standard of speaking of completing cleaning dishes should be much higher. Therefore, in the current study, we are interested in whether the effect of the interlocutor’s social status will also affect motion event endpoint description.

Clearly, there is an advantage of combining the effects of both the actor’s goal and the interlocutor’s status in motion description studies. However, rarely have any studies have done so, except for a recent study conducted by Liao et al. (2021). In their study, two Dutch directional prepositions (i.e., *naar* ‘to’ and *richting* ‘towards/direction’) have provided a nice paradigm for this. Specifically, they examined the extent to which the abovementioned two factors affected the choice between the two Dutch directional prepositions in a motion event description task. Before we discuss their study in more detail, we would like to first explain what directional prepositions are and why they make a nice paradigm for studying motion event endpoint description.

Directional prepositions, as part of a verb phrase, contribute to the telicity of an event—specifically, whether a motion event has an inherent endpoint or not. There are two types of directional prepositions: telic directional prepositions and atelic directional prepositions (Krifka, 1998; Zwarts, 2005). The use of telic directional prepositions, such as *naar* in Dutch and *to* in English, implies that a motion event is telic and has an inherent endpoint (e.g., *he is walking* ***to the bus station***). In contrast, the use of atelic directional prepositions, such as *richting* in Dutch and *towards* in English, only implies the direction of a motion event but not its endpoint. Therefore, a motion event that is described with an atelic directional preposition is considered lacking an inherent endpoint and is, therefore, atelic (e.g., *he is walking* ***towards the bus station***). Given the definition of directional prepositions and their classification, studying the choice between the two types of direction prepositions does provide a useful paradigm for us to explore the factors that could affect motion event endpoint description.

The current study is an extension of Liao et al. (2021). We aim to investigate the effects of the actor’s goal and the interlocutor’s status on the choice between a different pair of directional prepositions in a different language, that is, two English directional prepositions (i.e., *to* and *towards*) during motion event description. Furthermore, the current study also goes beyond Liao et al. (2021). Before we explain why this is the case, we would like to first provide a brief summary of Liao et al. (2021) and highlight the limitation of their study that we are about address in the current paper.

### A brief summary of Liao et al. (2021) and the limitation

Liao and colleagues (2021) adopted a motion event description task and examined the effects of the actor’s goal and the interlocutor’s status on the use of two Dutch directional prepositions (i.e., *naar* and *richting*).

They found that if the actor’s goal can be clearly inferred from the referential scenario, Dutch speakers use the telic preposition *naar* more often, compared with if no clear actor’s goal is presented in the scenario. For example, if the referential scenario depicts a man carrying a trash bag and a trash bin in the near distance, then his goal can be easily inferred from the scenario—that is, to go to the trash bin to dispose of the trash bag. However, if the referential scenario depicts a man carrying nothing and a trash bin in the near distance, the goal of the person is then not as clear as in the previous scenario. Consequently, Dutch speakers use *naar* more often (e.g., *hij loopt naar de container*—‘he walks to the trash bin’) when describing the first scenario to indicate the endpoint of the motion event is the trash bin, compared with when describing the second scenario. The opposite pattern is found for the use of the atelic preposition *richting*. That is, Dutch speakers use *richting* more often (e.g., *hij loopt richting de container—*‘he walks towards the trash bin’) for the second scenario than for the first scenario. This is because the use of *richting* does not indicate the reference object is the endpoint but just the actor’s moving direction.

Furthermore, they found that when the interlocutor is a police officer, Dutch speakers are more likely to use *richting* than when the interlocutor is a friend. This is because the social distance is larger between the speaker and the interlocutor if the interlocutor is a police officer, compared with if the interlocutor is a friend. When the social distance is larger, the speech context is more formal (Koppen et al., 2019). When the speech context is more formal, people also tend to be more specific and cautious with their statements. Given that *richting* only refers to the moving direction, not the endpoint of a motion event, the use of *richting* in a motion event description is considered a more conservative and more cautious expression compared with the use of *naar* because the speaker does not commit to a destination. Importantly, the effect of Interlocutor is also found larger than the effect of Intention (odds ratios: 1.72 vs. 3.79) in Liao et al. (2021), which highlights the importance of considering contextual factors in event description studies.

In Liao et al. (2021), only one verb form was used for all sentence stimuli—that is, the simple present tense (e.g., *Hij loopt naar/richting de container*—‘he walks to/towards the trash bin’). A possible effect of grammatical aspect is ruled out in their study, given that there is no grammaticalized progressive marker in Dutch (Flecken, 2011). The unmarked simple present is the major way to express ongoing events in Dutch. This is especially the case when ongoing directional motion events are described (e.g., *Hij loopt naar het station*—‘he walks to the station’; Liao et al., 2020; von Stutterheim et al., 2009).

However, the possible effect of grammatical aspect cannot be ruled out if an aspectual language such as English is studied. In English, the simple present is not the only way to express ongoing events. In fact, English has a grammaticalized progressive marker (i.e., -*ing*) that plays the main role in expressing ongoing events, including ongoing directional motion events (e.g., *he is walking to/towards the church*). As shown in many previous language-based event comprehension studies (e.g., Anderson et al., 2008; Ferretti et al., 2007; Madden & Zwaan, 2003; Matlock, 2011), grammatical aspect provides individuals with different viewpoints on the internal temporal structure of an event. When progressive aspect is used to express an event, more details about the event, especially the details relevant to the ongoing phase of the event, are activated during event comprehension, compared with when perfective aspect is used (e.g., Madden & Zwaan, 2003).

It is possible that the use of the English progressive aspect also creates a different event representation, compared with the use of the English simple present (e.g., *he walks to the trash bin* vs. *he is walking to the trash bin*). In the current study, we are specifically interested in whether the use of the simple present and the use of progressive aspect in English would result in people’s different sensitivity to the two factors that we are investigating, namely the actor’s goal and the interlocutor’s status. The more sensitive people are to these two factors, the larger effects they might create on people’s event endpoint description, which can be shown based on their choice between *to* and *towards*. Liao et al. (2021) did not investigate this possibility, which is a limitation of their study. Our current study, therefore, goes beyond the previous work by exploring the potential effect of grammatical aspect on event comprehension, and consequently, on event endpoint description.

In the next section, we provide a theoretical comparison between the English simple present and the English progressive aspect in their differences in representing ongoing events. A special focus will be put on how they can possibly affect the sensitivity to the two non-linguistic factors that we manipulate in the experiments.

### The simple present and progressive aspect in English

The simple present in English is often introduced in a dictionary as representing habits (e.g., *I smoke*), general truth (e.g., *he has long hair*), or even future events (e.g., *our meeting starts at 10:00 am*), and so on (see, for example, https://www.ef.com/wwen/english-resources/english-grammar/simple-present-tense/). In these cases, the English simple present is “timeless” (e.g., Vraciu, 2015). It takes a specific time reference from its linguistic environments, such as from the adverbial phrases (e.g., *at 10:00 am*), or it is used with a state (e.g., *he has long hair*), or it functions as a “stativizer” (Vraciu, 2015, p. 294) on dynamic predicates and creates a habitual reading of the dynamic event (e.g., *I smoke*).

The English simple present can also be used to describe dynamic ongoing events, such as in sports commentaries and narratives, without creating a habitual interpretation of these events. However, progressive aspect is still the major way of expressing ongoing events in English. Therefore, the question is what the differences are between the simple English present and the English progressive aspect when they are both used to describe ongoing events.

Many linguists have already provided some answers. One main difference is that the linguistic constructions might be different in representing event details. There is already much empirical evidence that the use of the English progressive aspect directs the comprehender’s attention to more event details, such as the location of the event (Ferretti et al., 2007), the intention of the actor (Sherrill et al., 2015), and the manner of action (Madden-Lombardi et al., 2017), compared with the use of perfective aspect.

The English simple present, however, often leads to a perfective interpretation of an event (see Cowper, 1998; Vraciu, 2015). When an event is viewed with a perfective viewpoint, this event is considered complete and “an unanalyzable whole” (Vraciu, 2015, p. 295). This perfective viewpoint provided by the English simple present is thus in contrast with the internal viewpoint provided by the English progressive aspect and is similar to the use of the English perfective aspect (i.e., -*ed*; Williams, 2002).

When the English simple present is used, for example, in sports commentaries (e.g., *Inzaghi passes the ball to Totti*; Williams, 2002, p. 1239), it represents the event (e.g., ‘to pass the ball’) in a holistic way, even though this event can still be happening at the moment of speech. By doing so, the speaker aims to push the whole story forward. Hence, sports commentaries in which the English simple present is used always consist of a series of events, for example, *Inzaghi passes the ball to Totti, he shoots, and the ball bounces off the goalpost* (Williams, 2002, p. 1241). It is odd to use the English progressive aspect here (i.e., *Inzaghi is passing the ball to Totti. He is shooting. The ball is bouncing off the goalpost). This is because the English progressive aspect emphasizes the progression of each event and places a spotlight on the ongoing phase of each event, which is part of an event instead of a whole event. This would affect the fluency of the development of the whole scenario/story.

Therefore, it is relevant to test whether the use of the English progressive aspect indeed creates a more salient representation of the internal temporal structure of an event, such as the actor’s manner, compared with when the English simple present is used. In the current study, the actor’s goal is indicated by the actor’s walking manner (e.g., holding a trash bag or not) in the referential scenario, which is relevant to the ongoing phase of the walking event. It is possible that the use of progressive aspect makes the participants more aware of the information relevant to the ongoing phase of the event (e.g., the actor’s goal/ the actor’s manner of walking) than when the English simple present is used. When the English simple present is used, the event is viewed as a whole, which might defocus the ongoing phase of the event and consequently, decrease the salience of our manipulation on the actor’s manner of walking (e.g., holding a trash bag or not) in the description task.

There is another difference between the English simple present and the English progressive aspect, when they are both used to describe ongoing events. When the English progressive aspect is used to report a scenario (e.g., *They’re all coming out of the front door. Two of them are wearing masks. One of them’s opening the car door and the others are getting in. They’re driving off now towards Friar Lane*; Williams, 2002, p. 1247), the unpredictable nature of the event is highlighted. The speaker is probably not sure about what is going to happen next and, therefore, chooses only to focus on describing the progression of the event. On the contrary, the English simple present is often used to report situations that are “rule-based,” “complete,” and “self-contained” (Williams, 2002, p. 1248), such as sports, ceremonies, or demonstrations. Under these circumstances, the speaker is not focusing on reporting an unpredictable scenario whose progression is important to the hearer. Instead, the speaker is trying to create an “eventful” scenario in which proper developments of complete events are presented.

Therefore, it is also important to test whether the use of the English progressive aspect will lead to a larger effect from the interlocutor’s status, compared with the use of the English simple present. It is possible that the use of the English progressive aspect makes the participants more aware of the speech context (i.e., to whom they are describing the scenario) than when the English simple present is used, given that the former highlights the unpredictable nature of an event, whereas the latter does not. For instance, when the speaker is aware that an event is still happening and its development is unpredictable, to whom they are asked to report this event could create a stronger effect on the cautiousness they have with their statements than when the speaker is not aware of the unpredictable nature of the event they are describing.

### The current study

The first goal of the current study is to investigate the effects of two non-linguistic factors (i.e., the actor’s goal and the interlocutor’s status) on the choice between two English directional prepositions (i.e., *to* and *towards*). Specifically, we are interested in whether the observed effects of the actor’s goal and the interlocutor’s status in Liao et al. (2021) are upheld when another language is studied. English is chosen because English and Dutch are different in their aspectual systems. This relates to the second goal of our study, which is to investigate the effect of one linguistic cue, grammatical aspect, on the sensitivity to the two non-linguistic factors, and consequently on motion event endpoint description. This second goal of our study goes beyond Liao et al. (2021), in that in their study, the effect of grammatical aspect was not considered.

We combine both comprehension and production tasks in the current study. The comprehension task targets the comprehension of the simple present and progressive aspect used in the sentence stimuli (e.g., *he walks/is walking (?) the trash bin*). At the same time, participants performed a description task by choosing between *to* and *towards* to complete the sentence stimuli (e.g., *he walks* (? *to/towards) the trash bin*).

In Experiment 1, we used the simple present for all our sentence stimuli (e.g., *he walks (?) the trash bin*). This is a reasonable start, given that our first goal is to extend the findings in Liao et al. (2021; i.e., the main effects of the actor’s goal and the interlocutor’s status on preposition choice during motion event description). By using the English simple present in Experiment 1, we managed to keep the surface form of the verbs of our sentence stimuli (e.g., *he walks (?) the trash bin*) the same as those in Liao et al. (2021). Our hypothesis for Experiment 1 is that there should be the main effects of both the actor’s goal (Intention) and the interlocutor’s status (Interlocutor) on the choice between *to* and *towards* in the event description task. At the same time, we are aware of the possibility that the use of the English simple present might weaken the effects of the two factors.

In Experiment 2, we replaced the English simple present with the English progressive aspect for all the sentence stimuli (e.g., *he is walking (?) the trash bin*). Even though we have discussed the possible differences between the English simple present and the English progressive aspect in affecting the sensitivity to the two factors (i.e., Intention and the Interlocutor) that we manipulate in the description task, these lines of thought are still speculative. To our knowledge, in addition to the above-discussed linguistic analyses, no experimental studies have been published to date on comparing the English simple present to the English progressive aspect regarding their role in representing event details, let alone from the perspectives of studying the actor’s goal and the interlocutor’s status. Hence, we decided to formulate two hypotheses for Experiment 2:

#### Hypothesis 1

The English progressive aspect does not differ from the English simple present in affecting people’s sensitivity to the two factors that we manipulate in the description task (i.e., Intention and Interlocutor). The effects of the two factors (on the use of *to* and *towards*) that we obtain in Experiment 2 should be the same as those we found in Experiment 1.

#### Hypothesis 2

The English progressive aspect does differ from the English simple present in affecting people’s sensitivity to the two factors (i.e., Intention and Interlocutor). The English progressive aspect should make people more sensitive to the two factors in the description task, compared with the English simple present. We should find the main effects of both Interlocutor and Intention (on the use of *to* and *towards*), and the effects of both factors should be larger than those found in Experiment 1.

### Frick’s COAST method and sequential testing

As in Liao et al. (2021), we adopted Frick’s COAST method for both experiments conducted in the current study. This method preserves an overall alpha level of .05 while allowing for sequential testing (Frick, 1998). There are many advantages for researchers to choose sequential analyses over a conventional fixed-sample testing method (see Lakens, 2014). One major advantage is that sequential analyses can greatly help researchers to run sufficiently powered studies without running an inefficiently large number of participants. Determining a fixed sample for a high-powered study is not easy and often faces much uncertainty. If the determined sample size is too small, the study has the risk of being underpowered and the obtained effect size is often inaccurate. On the other hand, if the sample size is much bigger than actually needed, it is a waste of time, resources, and energy. Sequential analyses can be used for testing larger samples and at the same time allow for earlier termination of data collection. This increases the statistical power of the study and also prevents researchers from wasting participants. Moreover, given the adjusted lower alpha level, if data collection is stopped earlier with a relatively small sample size, the estimated effect size is still more reliable than a traditional small-scale study (Lakens, 2014, p. 703).

## Experiment 1

Experiment 1 was designed to extend the findings reported in Liao et al. (2021). Based on their findings, we hypothesized the main effects of both the actor’s goal and the interlocutor’s status in this experiment. Except that we replaced the scenarios that contained a man and a bike repair shop with a more distinct version (see detailed clarification in the Materials section and in the preregistration: https://osf.io/7c5zh/?view_only=54cdbbb89cfb4f58a952edf8bd7331ab), the design, the data collection plan, and the data analysis plan were all kept the same as those of the second experiment in Liao et al. (2021). As in Liao et al. (2021), we also used the simple present in Experiment 1 for all the sentence stimuli (e.g., *he walks (?) the trash bin*). The whole experiment was in English, including the instructions and the sentence stimuli.

### Method

#### Participants

Frick’s COAST (Frick, 1998) method was adopted for data collection and for conducting sequential analyses. Specifically, we recruited participants in batches of 160 participants (i.e., the minimum number of participants to be tested; 80 per Intention and 80 per Interlocutor). We predicted the main effects of Intention (the actor’s goal) and Interlocutor (the interlocutor’s status). If *p* < .01 or *p* > .36 for both the main effects we predicted, data collection would be terminated. If *p* was within these boundaries for any one of the two main effects predicted, we would test another 160 participants. Data collection would be terminated if the number of participants reached 480, regardless of the *p* values at that time. We recruited 480 participants (315 males, 164 females, one other; mean age 34.77 years old, range: 20-74 years old) eventually. All participants reported their native language as English. Participants were recruited via Mechanical Turk. The experiment took around 1 to 2 minutes per participant and each participant was paid $0.50 as a reward.

#### Materials

**Fig. 1:**
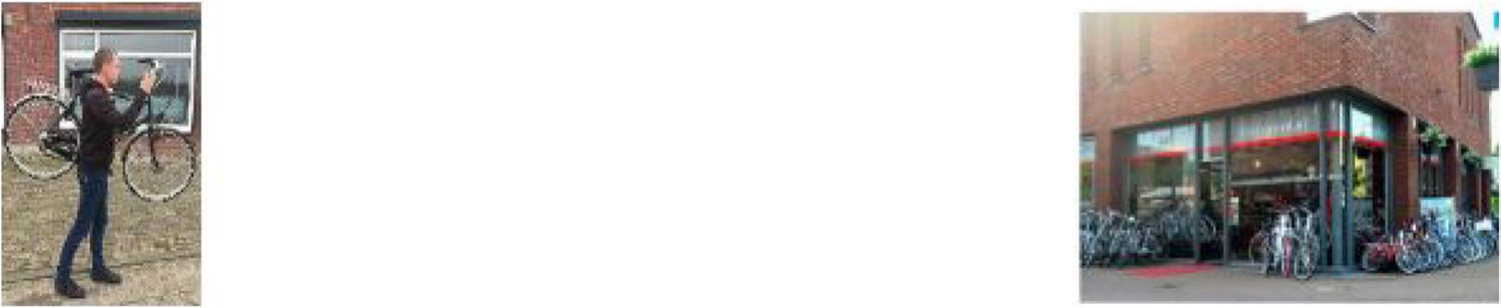
An example of the bike scenarios used in Liao et al. (2021)

**Fig. 2:**
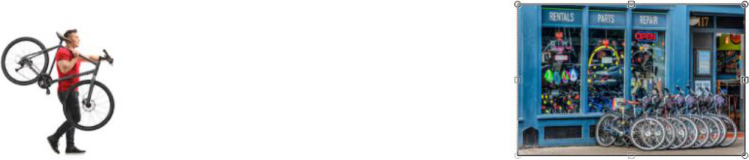
An example of the bike scenarios used in the current study

The experiment was programmed using the Qualtrics Survey Software. As in Liao et al. (2021), we used two different scenarios (one was with a person and a bike repair shop and the other one was with a person and a trash bin). For each scenario, there were two versions. In the first scenario, the person either carried a broken bike or not; in the second scenario, there was a person who either carried a trash bag or not. Therefore, there were in total four scenarios as the experimental stimuli (see Appendix). The trash bin scenarios were exactly the same as those used in Liao et al. (2021). We replaced the bike scenarios in Liao et al. (2021) with their more distinct versions (see an example of the ones used in their study and its replacement that was used in the current study in Figs. 1 and 2, respectively). We did so because we wanted to have a homogeneous layout for both the bike repair shop and the trash bin scenarios, for example, no background in the picture of the person. Moreover, we thought that if there was no background in the picture of the person, it would be easier for participants to combine the two pictures—for instance, the person and the bike repair shop—into one holistic scenario. The bike repair shop was also replaced with one with signs on it (e.g., repairs, rental) to ensure that participants knew it was a bike repair shop. Below each picture, a sentence was shown (e.g., *he walks (?) the bike repair shop*) and a choice between *to* and *towards* was shown below the sentence (see Appendix).

#### Design and procedure

Sixteen cells were designed (4 Scenario [Intention] × 2 Interlocutor × 2 OptionOrder) and each participant was randomly assigned to only one cell. We manipulated the instructions as either “*You are describing a scenario to a police officer as a witness*” or “*You are describing a scenario to a friend*” (Interlocutor). Progressive aspect was used in the instructions because we wanted the participants to imagine their interlocutor as vividly as possible while performing the description task. The choice option order was counterbalanced (*to* at the right side of *towards* or at the left side of *towards*). An informed consent form was attached at the beginning of the survey. Participants could choose whether to continue the survey or to leave freely at any time. Then they answered questions about their demographic information (i.e., gender and age) and their mother tongue. They subsequently read the instructions of the experiment on the screen and chose between *to* and *towards* to complete the sentence stimuli, based on the scenario they were viewing.

### Results and discussion

#### Confirmatory analyses

A binomial logistic regression model that included the main effect of Intention and the main effect of Interlocutor was conducted in R (R Core Team, 2016) using the *glm* function implemented in the package *lme4* (Bates et al., 2015; the formula used in R was *preposition ~ intention + interlocutor*). Both factors were dummy coded. No interaction effect was included in the model. The dependent variable was the directional preposition choice, which was a binary outcome. The choice of the directional preposition *to* was coded as “0” and the choice of the directional preposition *towards* was coded as “1” in R (in alphabetic order). In the first batch of collected data (*N* = 160), we found a significant main effect of Intention (*β* = −0.995, *SE* = 0.34, *z* = −2.919, *p* = .004, odds ratio: 0.370, 95% CI [0.19, 0.72]). We did not find a significant main effect of Interlocutor but the *p* value was within the boundary from .01 to .36 (*β* = 0.676, *SE* = 0.34, *z* = 1.989, *p* = .047, odds ratio: 1.965, 95% CI [1.02, 3.86]).

Given that the *p* value found for the effect of Interlocutor was within the boundary from .01 to .36, we continued data collection. After the second round of data collection (*N* = 320), we performed the same analysis. We found that both the main effect of Intention and the main effect of Interlocutor were not significant (*Intention β* = −0.482, *SE* = 0.23, *z* = −2.071, *p* = .038, odds ratio: 0.62, 95% CI [0.39, 0.97]; *Interlocutor β* = 0.534, *SE* = 0.23, *z* = 2.296, *p* = .022, odds ratio: 1.71, 95% CI [1.08, 2.70]). The *p* values for both factors were within the boundary from .01 to .36. Therefore, we continued data collection until we reached 480 participants. Based on our preregistration, this was our last round of data collection.

**Fig. 3:**
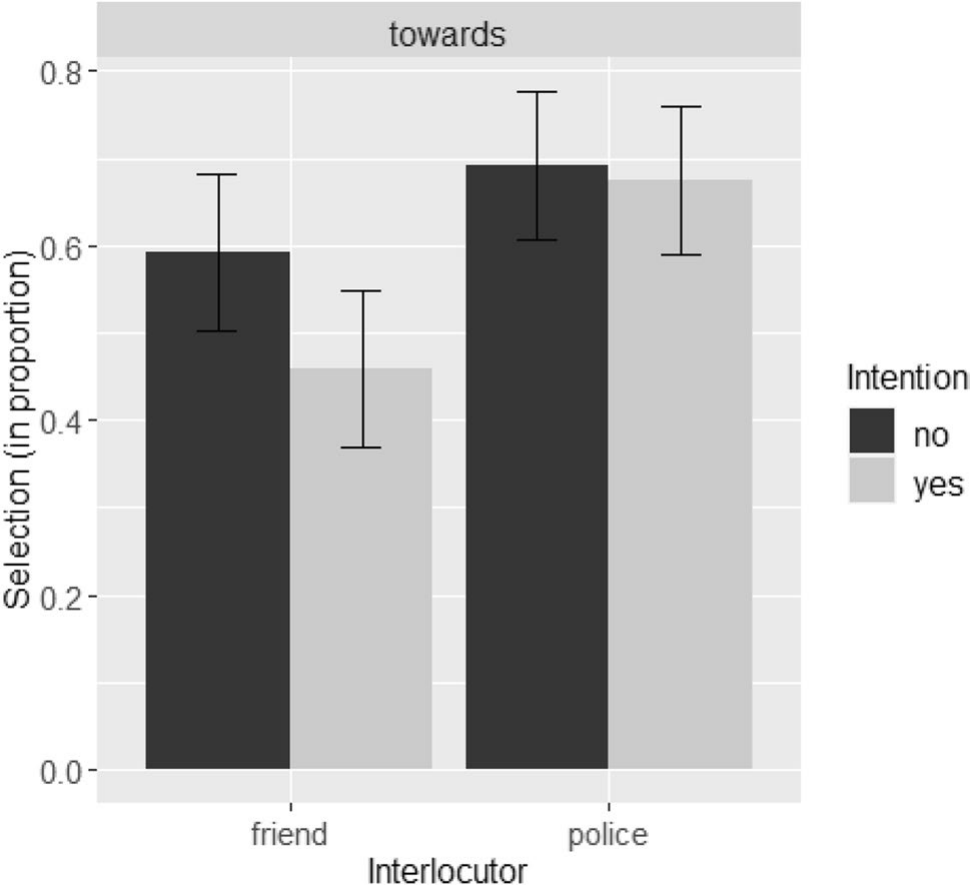
Mean proportions of the selection of *towards* under the conditions of Intention and Interlocutor in Experiment 1. Error bars present 95% confidence intervals for the mean

We did the same analysis again for the last data batch (*N* = 480). We did not find a significant effect of Intention (*β* = −0.323, *SE* = 0.19, *z* = −1.700, *p* =.089, odds ratio: 0.72, 95% CI [0.50, 1.05]). The *p* value of the main effect of Interlocutor, however, was smaller than .01 (*β* = 0.673, *SE* = 0.19, *z* = 3.538, *p* < .001, odds ratio: 1.96, 95% CI [1.35, 2.85]): based on the standards of the sequential analysis we pre-registered, it was considered a significant effect. Thus, we found support for the hypothesis that addressee status affects preposition choice but not for the hypothesis that protagonist intention does the same. Figure 3 shows the mean proportions of the selection of *towards* under the conditions of Intention and Interlocutor in Experiment 1.

#### Exploratory analyses

The interaction effect between Intention and Interlocutor was not considered when forming our hypotheses in the confirmatory analyses. It was possible that different interlocutors might affect the sensitivity to the actor’s goal. However, we were not sure in what direction Interlocutor might affect the sensitivity to the two levels of Intention (i.e., a clear goal vs. an unclear goal). One possibility was that talking to a police officer might lower participants’ willingness to commit themselves to an event endpoint no matter whether the actor’s goal was clear or unclear, compared with when talking to a friend. Another possibility was that talking to a police officer would only lower the certainty about an event endpoint when the actor’s goal was not clear but would not make much difference when the actor’s goal was clear, compared with when talking to a friend. In such circumstances, we would like to explore these possibilities in the exploratory analyses.

Apart from the interaction between Intention and Interlocutor, we also decided to add the scenario type as a fixed effect in the exploratory analyses (including its main effect and its interaction effect with Intention and with Interlocutor). Scenario type was supposed to be taken as a random effect in the confirmatory analyses. However, the inclusion of it as a random effect in the confirmatory analyses brought overfitted warnings. Moreover, the scenario type had only two levels (i.e., trash bin vs. bike repair shop). When a random factor has too few levels (normally fewer than five levels; Bolker, 2015), the estimated variance can be very imprecise and unstable, especially when a singular fit warning occurs (Oberpriller et al., 2021). Under such circumstances, researchers recommend fitting such a random effect in the statistical model as a fixed effect (Bolker, 2015; Crawley, 2002; Gelman, 2005; Gelman & Hill, 2007).

Moreover, we were interested in whether there was a main effect of the order of the two prepositions as an option. We counterbalanced this factor in our experimental design to avoid a possible primacy or recency bias in the participants’ answers. That is, participants might prefer to choose the first option they encountered (a primacy bias) or on the contrary, participants might be more likely to choose the latest option they saw (a recency bias). In the explanatory analyses, we were interested in whether such biases indeed existed in our experiments or not.

Therefore, we built a binomial logistic regression model that included the main effects of Intention, Interlocutor, Option Order, Scenario, the interaction between Intention and Interlocutor, the interaction between Scenario and Intention, and the interaction between Scenario and Interlocutor (the formula used in R was *preposition ~ intention × interlocutor + intention × scenario + interlocutor × scenario + option order*). All the factors were sum coded except for the factor Option Order (dummy coded), for we were only interested in the main effect of this factor. The dependent variable was the choice between the two directional prepositions: *to* and *towards*. The choice of *to* was coded as “0” and the choice of *towards* was coded as “1.”

The main effect of Intention was still not significant (*β* = 0.164, *SE* = 0.10, *z* = 1.666, *p* =.096, odds ratio: 1.18, 95% CI [0.97, 1.43]). The significant main effect of Interlocutor (*β* = −0.374, *SE* = 0.10, *z* = −3.759, *p* <.001, odds ratio: 0.69, 95% CI [0.57, 0.84]) remained in this model. No interaction effect was found between Intention and Interlocutor (*β* = 0.106, *SE* = 0.10, *z* = 1.082, *p* = .28, odds ratio: 1.11, 95% CI [0.92, 1.35]). We found a significant main effect of Scenario (*β* = −0.377, *SE* = 0.10, *z* = −3.786, *p* <.001, odds ratio: 0.69, 95% CI [0.56, 0.83]). A significant interaction effect between Scenario and Interlocutor (*β* = 0.273, *SE* = 0.10, *z* = 2.748, *p* =.006, odds ratio: 1.31, 95% CI [1.08, 1.60]) was also detected. Specifically, *towards* was used more often when the addressee was a police officer than when it was a friend, only for the scenarios of trash bins. No interaction effect was found between Scenario and Intention (*β* = −0.064, *SE* = 0.10, *z* = -0.647, *p* =.52, odds ratio: 0.94, 95% CI [0.77, 1.14]). There was no significant main effect of Option Order (*β* = 0.151, *SE* = 0.19, *z* = 0.776, *p* = .438, odds ratio: 1.16, 95% CI [0.80, 1.70]).

In sum, we predicted the main effects of Intention and of Interlocutor on the use of *to* and *towards* in Experiment 1. In the confirmatory analyses, we did find a main effect of Interlocutor (*p* < .001, odds ratio: 1.96), but not a main effect of Intention (*p* = .089, odds ratio: 0.72; this was also confirmed in the explanatory analyses). Moreover, the effect sizes of both factors (indicated by their odds ratios: Intention vs. Interlocutor: 1.39 vs. 1.96) were lower than those found in Liao et al. (2021) (Intention vs. Interlocutor: 1.72 vs. 3.79). According to our previous discussions (see the section *The simple present and progressive aspect in English*), a possible reason is that the use of the English simple present might have weakened the salience of both factors, Intention and Interlocutor, in Experiment 1. The use of the English progressive aspect might help to increase the salience of both factors and hence also strengthen their effects on preposition choice. Therefore, in Experiment 2, we replaced the English simple present with the English progressive aspect for all the sentence stimuli to examine this possibility.

## Experiment 2

Experiment 2 was identical to Experiment 1, except that we replaced the English simple present used in the sentence stimuli (e.g., *he* ***walks*** *(?) the trash bin*) with the English progressive aspect (e.g., *he* ***is walking*** *(?) the trash bin*). We formed alternative hypotheses for Experiment 2 (see The Current Study section).

### Method

#### Participants

As in Experiment 1, we adopted Frick’s COAST method for data collection and for conducting sequential analyses. We also recruited participants in batches of 160 participants. If *p* < .01 or *p* > .36 for both factors (Intention and Interlocutor), data collection would be terminated. If *p* was within these boundaries for any one of the two factors, we would test another 160 participants. Data collection would be terminated if the number of participants reached 480, regardless of the *p* values at that time. In the end, we ended data collection at our first data batch (*N* = 160, 83 males, 73 females, four others; mean age 33.675 years old, range: 17–74 years old), given that the *p* values for both factors were below .01 when *N* = 160. All participants were native English speakers and were recruited via the Prolific platform. The experiment took around 2 minutes per participant and each participant received £0.35 as a reward.

#### Materials

The same materials as in Experiment 1 were used, except that the simple present used in Experiment 1 was replaced with progressive aspect for all the sentence stimuli.

#### Design and procedure

The design and procedure were identical to Experiment 1, except that participants were not asked to fill in information about their mother tongue. This is because we already excluded people whose first language was not English through the Prolific platform, and it was not allowed to collect information about people’s linguistic backgrounds on this platform.

### Results and discussion

#### Confirmatory analyses

**Fig. 4:**
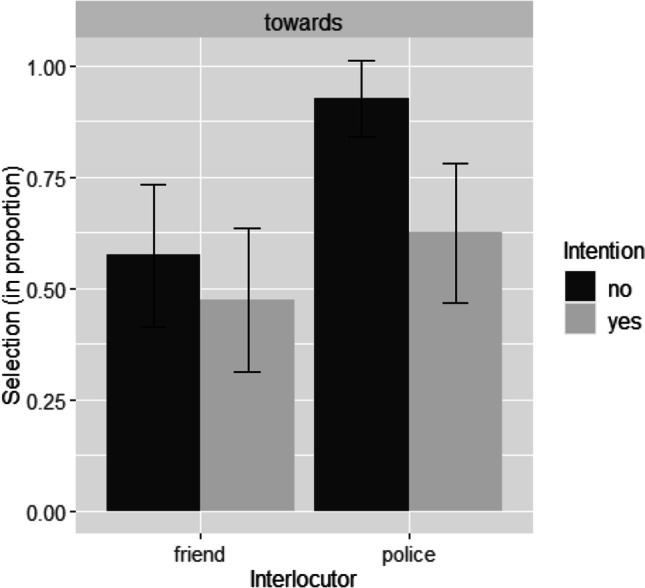
Mean proportions of the selection of *towards* under the conditions of Intention and Interlocutor in Experiment 2. Error bars present 95% confidence intervals for the mean

The same binomial logistic regression model that included the main effect of Intention and the main effect of Interlocutor was conducted in R using the *glm* function implemented in the package *lme4* (Bates et al., 2015; the formula used in R was *preposition ~ intention + interlocutor*). Both factors were dummy coded. No interaction effect was included in the model. The dependent variable was the directional preposition choice, which was a binary outcome. The choice of *to* was coded as “0” and the choice of *towards* was coded as “1” in R (in alphabetic order). We found significant main effects of both Intention (*N* = 160; *β* = −0.969, *SE* = 0.36, *z* = −2.711, *p* = .007, odds ratio: 0.379, 95% CI [0.19, 0.76]) and Interlocutor (*β* = 1.195, *SE* = 0.36, *z* = 3.322, *p* < .001, odds ratio: 3.31, 95% CI [1.66, 6.82]). Figure 4 shows the mean proportions of the selection of *towards* under the conditions of Intention and Interlocutor in Experiment 2.

#### Exploratory analyses

As in Experiment 1, we built the same binomial logistic regression model that included the main effects of Intention, Interlocutor, Option Order, Scenario, the interaction between Intention and Interlocutor, the interaction between Scenario and Intention, and the interaction between Scenario and Interlocutor (the formula used in R was *preposition ~ intention × interlocutor + intention× scenario + interlocutor × scenario + option order*). All the factors were also sum coded except for the factor Option Order (dummy coded), for we were only interested in the main effect of this factor. The reasons why we included these factors in the exploratory analyses were already provided in the *Exploratory analyses* section in Experiment 1.

Both the main effects of Intention and Interlocutor remained significant in this model (*Intention β* = 0.623, *SE* = 0.21, *z* = 2.969, *p* = .003, odds ratio: 1.86, 95% CI [1.26, 2.92]; *Interlocutor β* = −0.732, *SE* = 0.21, *z* = −3.471, *p* < .001, odds ratio: 0.48, 95% CI [0.31, 0.71]). The interaction between Intention and Interlocutor was found to be marginally significant if we adopted the conventional rule of determining a significant effect when *p* < .05 (*β* = −0.419, *SE* = 0.21, *z* = −1.998, *p* = .046, odds ratio: 0.66, 95% CI [0.42, 0.97]). Specifically, *to* was used less frequently when the actor’s goal could not be clearly inferred from the scenario, compared with when it could, particularly so when the interlocutor was a police officer, compared with when the interlocutor was a friend. The main effect of Scenario was not significant (*β* = 0.089, *SE* = 0.20, *z* = 0.455, *p* = .649, odds ratio: 1.09, 95% CI [0.75, 1.62]), and neither was its interaction with Intention (*β* = 0.079, *SE* = 0.19, *z* = 0.411, *p* = .681, odds ratio: 1.08, 95% CI [0.75, 1.59]) and with Interlocutor (*β* = −0.292, *SE* = 0.20, *z* = −1.501, *p* = .133, odds ratio: 0.75, 95% CI [0.50, 1.09]). The main effect of Option Order was also found insignificant (*β* = 0.255, *SE* = 0.36, *z* = 0.713, *p* = .476, odds ratio: 1.29, 95% CI [0.64, 2.62]).

These findings support the second hypothesis formed in Experiment 2. We found main effects of both Intention and Interlocutor. Their effect sizes (indicated by the odds ratios: Intention vs. Interlocutor: 2.64 vs. 3.31) were also larger than those found in Experiment 1 (Intention vs. Interlocutor: 1.39 vs. 1.96; from the confirmatory analyses). Therefore, we conclude that the use of the English progressive aspect indeed leads people to pay more attention to event details and to speech context, compared with when the English simple present is used.^1^

## General discussion

In the current study, two experiments were conducted to examine the effects of two non-linguistic factors, namely the actor’s goal and the interlocutor’s social status, on motion event endpoint description. Moreover, the effect of one linguistic cue, grammatical aspect, on the sensitivity to the two non-linguistic factors was also investigated. By examining the choice between two Dutch directional prepositions (i.e., *naar* and *richting*), a study conducted by Liao et al. (2021) demonstrates that both the actor’s goal and the interlocutor’s status affect motion event endpoint description. Our first goal here was to extend these findings by investigating the English equivalents of the two Dutch prepositions—namely, *to* and *towards*. Our second goal was to go beyond their study by studying whether different grammatical aspect (i.e., the English simple present and the English progressive aspect) would affect the salience of the two non-linguistic factors (i.e., Intention and Interlocutor) in the description task and consequently also affect people’s motion event endpoint description.

In Experiment 1, we used the English simple present for all the sentence stimuli (e.g., *he walks to/towards the bike repair shop*). We predicted the same significant main effects of both Intention and Interlocutor as in Liao et al. (2021). We did find a significant effect of Interlocutor (odds ratio: 1.96). Its effect size, however, was almost twice as small as that found in Liao et al. (2021) (odds ratio: 3.79). Moreover, we did not find a significant effect of Intention in Experiment 1 (*p* = .089, odds ratio: 0.72).

We assume that the insignificant effect of Intention and the smaller effect of Interlocutor (compared with the one in Liao et al., 2021) should be attributed to the use of the English simple present in Experiment 1. As previously mentioned, although the English simple present can be used to express ongoing events, it does not emphasize the progressive phase of the event. Instead, it tends to present the event as a whole and defocuses the internal temporal structure of the event. In our experiments, the actor’s goal was indicated by an object the actor was or was not carrying (e.g., carrying a trash bag or not), which was relevant to the progressive stage of the actor’s action. For instance, if the actor was carrying a trash bag, it was then clearer that the actor was to go to the trash bin to dispose of the trash bag, than when the actor was carrying nothing. However, when an event was viewed as a unified whole (an external viewpoint provided by the use of the English simple present: *walks*), the ongoing phase of an event, including the actor carrying or not carrying an object, was then defocused. Consequently, participants in Experiment 1 became less sensitive to the factor Intention when performing the description task. In Liao et al. (2021), the effect of Intention was significant but not particularly strong (odds ratio: 1.72). Hence, it is reasonable that this effect was absent in Experiment 1 in the current study.

The use of the English simple present also led to a decreased sensitivity to the speech context. As we have discussed previously, the use of the English simple present imbues an event with a more predictable and more preplanned sense, than when the English progressive aspect is used. This is because the English simple present does not emphasize the progression of an event and what is exactly happening at the moment of speech, but instead focuses on a more complete and holistic presentation of the event. When an event is more predictable, the speech context could then be comprehended as less important, than when an event is presented as less predictable. This, however, does not necessarily cause the disappearance of the effect of speech context if its effect is strong enough. The effect size of Interlocutor was indeed fairly large in Liao et al. (2021). Therefore, we found that the effect of Interlocutor was also present in Experiment 1 but was smaller than the effect found in Liao et al. (2021).

In Experiment 2, we replaced the English simple present with the English progressive aspect for all the sentence stimuli, and we found the main effects of both Intention and Interlocutor on the use of *to* and *towards*. Moreover, the effect sizes of both factors found in Experiment 2 were larger than those found in Experiment 1. These findings support our second hypothesis formed in Experiment 2. The English progressive aspect indeed brings about a more careful reading of both the actor’s goal and the interlocutor’s status during event comprehension, compared with the English simple present.

Therefore, an important conclusion we can draw based on the results of these experiments is that the effects of the actor’s goal and the interlocutor’s status on motion event description that were found in Liao et al. (2021) are indeed stable, given that those effects were also detected among English native speakers when *to* and *towards* were investigated. Our study confirms the idea that during the process of event description, knowing the actor’s goal is essential to the identification of an event endpoint. This is not surprising, given that humans are intentional agents whose behaviors are normally goal directed (see also Zacks & Swallow, 2007). Moreover, a goal is set to be achieved. Where there is a goal, there is an expected endpoint.

Our finding of the role of the actor’s goal in identifying an event endpoint during event description is similar to what Zacks has proposed about the role of the actor’s goal in event segmentation (see Zacks, 2004). As put forward by Zacks (2004), the actor’s goal is one of the defining features of the “knowledge structures for events” (p. 980). It works as a cue for detecting an event boundary in a top-down manner during the process of ongoing-activity segmentation. Similarly, we assume that how the actor’s goal affects event endpoint description is also a sort of top-down processing.

Moreover, the actor’s goal is an internal feature. Most of the time, it needs to be explicitly expressed or inferred from the movements of the actor (Radvansky & Zacks, 2014; Zacks, 2004). Our study demonstrates that people indeed make use of the information provided in the referential scenarios, including the actor carrying or not carrying an object, to get access to the intention of the actor for predicting an event endpoint during event description. This is bottom-up processing since the actor’s goal is inferred from the sensory information presented in the referential scenarios. This bottom-up processing is incorporated with subsequent top-down processing (i.e., inferring an event endpoint from the actor’s goal) during the whole event description phase.

Our study also confirms that speech context, such as the social distance between the speaker and the interlocutor, plays an important role in event endpoint description. Previous studies have demonstrated that people adapt their speech behavior depending on the formality of the speech context. They found that people use nouns, prepositions, and adjectives more frequently in a more formal speech context, compared with in a less formal speech context (e.g., Heylighen & Dewaele, 2002; Koppen et al., 2019). Our study focuses on the social status of the interlocutor, which is one important parameter that determines the formality of the speech context. We found that even the choice between specific prepositions differs depending on the social distance between the speaker and the interlocutor. Specifically, people use a more specific preposition (e.g., *towards*) to define an event more often if they talk to a formal interlocutor, such as a police officer, compared with if they talk to an informal interlocutor, such as a friend.

Importantly, the effect of Interlocutor was found to be larger than the effect of Intention on motion event endpoint description. There are two possible reasons for this. First, Interlocutor is a macro feature about the context that has general importance for a speaker during an event description task. Therefore, when information about the speech context is clarified, the speaker is ready to take this information on board and make use of it immediately. However, Intention is a specific event component. Speakers usually do not presuppose that they need this information when describing an event. Therefore, during the description tasks, participants should pay less attention to protagonist intention than to interlocutor status. Second, during the description task, the interlocutor information was expressed explicitly through linguistic expressions, whereas the intention information had to be inferred from the picture. The former should be more straightforward and easier to process, compared with the latter. These factors might have cumulative effects, which explains why the effect of Interlocutor was found to be larger than the effect of Intention.

Hence, our study highlights the urgency of considering contextual factors in event description studies, which are currently understudied in this field. The role of the interlocutor’s social status in event endpoint description also sheds light on eyewitness testimony studies. For example, police officers should be aware that their identity might create an unconscious effect on how their witnesses describe a crime. Witnesses might become more careful and more conservative with their language use than they normally do, which is not always helpful for solving cases, especially if they do not dare to commit themselves to any certain statements.

Furthermore, our study demonstrates that grammatical aspect influences people’s mental representations of event details and even people’s sensitivity to speech context. This is an important message for both event comprehension and event production studies, given that even a small difference in the use of grammatical aspect (i.e., the simple present vs progressive aspect) can lead to different representations of the depicted event. For event production studies, it is, hence, important to take into account any possible effects that might come from the verb forms used in the instructions or in any other experimental materials.

### Limitations of the current study and future research

For both experiments in the current study, we used only two types of scenarios (i.e., the trash bin scenarios and the bike shop scenarios). This is mainly because for our study, it was very important to ensure that participants did not know the goal of the experiments. Their preposition choice should be based on their linguistic intuition without any awareness of our manipulations. Otherwise, their responses would become useless. To avoid participants from knowing the goal of our experiments, we adopted a between-subjects design. In this design, we assigned one participant only one cell out of the total 16 cells (2 Scenario*×* 2 Intention*×* 2 Interlocutor*×* 2 OptionOrder). Each cell was assigned to 10 participants for the first data batch, based on our preregistration plan. If we had added one more scenario, we would have to create eight more cells (3 Scenario*×* 2 Intention*×* 2 Interlocutor*×* 2 OptionOrder: 24 cells). Consequently, we would have needed to recruit at least 80 more participants, which we consider a waste of resources and was not worthwhile to do so.

However, we are aware of the generalizability issue due to the limited number of scenarios used in the current study. We are positive that our findings can be generalized to other scenarios. One main reason is that the two chosen types of scenarios represent two common motion events in daily life. They are not special regarding the nature of the motion events they represent but they still represent two unrelated motion events. Given that the effects of the protagonist intention and the interlocutor’s status have already been generalized across these two common but unrelated scenarios in two different languages, we are confident that these effects can be generalized to other types of scenarios. Moreover, the concepts of the two studied factors (i.e., Intention and Interlocutor) and the use of grammatical aspect are not limited to the characteristics of the two chosen scenarios. It is, therefore, feasible to consider other scenarios to manipulate these factors.

What should be noted is that possible differences between scenarios might affect the strength of the effects of these factors on endpoint conceptualization. As we can infer from the explanatory analyses of our Experiment 1, when the sensitivity to the interlocutor’s status was weakened by the use of the English simple present, its effect was only detected in the trash bin scenarios, not in the bike shop scenarios. This indicates a possible difference between the trash bin scenarios and the bike shop scenarios in relation to the effect of Interlocutor, even though this difference eventually disappeared in Experiment 2 when the English progressive aspect was used. Hence, we do not formulate strong claims here regarding the specific scenarios that our findings can generalize to but leave that for future research. When designing scenarios for future research, researchers are recommended to perform a norming study to have a clearer idea of how the scenarios differ in the degree of certainty that the referred location is the destination of the moving entity. Future research should also explore more types of motion events and that may even go beyond the scope of motion events. If possible, a more naturalistic depiction of events, for instance, using videos of events, is also recommended.

Another limitation of the current study is that we did not take a possible interaction effect between the actor’s goal and the interlocutor’s status into account in the preregistered analyses. As shown in the explanatory analyses of Experiment 2, however, there was a marginally significant interaction between the two factors. Specifically, when talking to a police officer, participants were especially less willing to commit themselves to an event endpoint when the actor’s goal was unclear (compared with when the actor’s goal was unclear), compared with when talking to a friend. However, the effect size of this interaction was relatively small (odds ratio: 1.52). Moreover, detecting a reliable interaction effect often requires a larger sample size than detecting a main effect. Given that we did not plan our sample size for finding an interaction, we are uncertain whether the detected marginally significant interaction between the two factors is a true effect or is just a positive false. Theoretically speaking, it is indeed possible that people show different sensitivity to the actor’s goal depending on to whom they are talking. Therefore, future research should consider this interaction effect when conducting event endpoint conceptualization studies.

### Conclusion

The current study extends the findings reported by Liao et al. (2021). Our findings support the idea that both the actor’s goal and the interlocutor’s status affect motion event endpoint description, even when speakers with a different native language were tested. Our study contributes to motion event description studies by providing evidence that the absence/presence of a clear intention of the actor is an important factor in event endpoint description. Moreover, our study highlights the importance of considering contextual factors, such as the social status of the interlocutor, in event description studies.

Importantly, our study provides further evidence that grammatical aspect (i.e., the English simple present and the English progressive aspect) also affects event endpoint description, via their influence on event details representation and the perception of speech context. Unlike most event representation studies that focus on the difference between the English progressive aspect and the English perfective aspect, the current study provides a novel perspective in event representation studies—that is, including the contrast between the English simple present and the English progressive aspect. Many linguists have theoretically analyzed their difference in representing eventualities. However, to our knowledge, no studies have experimentally tested this difference. The current study provides experimental evidence for their different role in event representation. A take-home message here is that subtle differences in language use, such as the use of different verb forms, can result in a substantial change in meaning.

## Footnotes

1 A reviewer mentioned the possibility of a direct effect of aspect on preposition choice, which means a possibility of more *towards* phrases in sentences with a progressive aspect compared with *to* phrases. We excluded this possibility through corpus search in Sketch Engine (https://www.sketchengine.eu/). We first searched the number of hits for the phrases ***walking to***, ***walking toward*** and ***walking towards*** in the whole corpus provided in Sketch Engine. We then filtered out the instances that had verbs at either the Right 1 or the Right 2 positions of the prepositions *to*, *toward*, or *towards*. We found that the number of hits of ***walking to*** (41,955) was 1.6 times higher than that of both ***walking toward*** and ***walking towards*** (in total 26,041).
